# Saffron: A Multitask Neuroprotective Agent for Retinal Degenerative Diseases

**DOI:** 10.3390/antiox8070224

**Published:** 2019-07-17

**Authors:** Stefano Di Marco, Veronica Carnicelli, Nicola Franceschini, Mattia Di Paolo, Marco Piccardi, Silvia Bisti, Benedetto Falsini

**Affiliations:** 1 Department of Applied Clinical Science and Biotechnology, University of L’Aquila, Via Vetoio, Coppito II, 67100 L’Aquila, Italy; 2 Istituto Nazionale di Biostrutture e Biosistemi (INBB), Via Medaglie d’Oro 305, 00136 Roma, Italy; 3 Facolta’ di Medicina e Chirurgia, Fondazione Policlinico A. Gemelli, Universita’ Cattolica del S. Cuore, 00136 Roma, Italy

**Keywords:** oxidative stress, age related macular degeneration, light induced damage, matrix metalloproteinases, saffron

## Abstract

Both age related macular degeneration (AMD) and light induced retinal damage share the common major role played by oxidative stress in the induction/progression of degenerative events. Light damaged (LD) rats have been widely used as a convenient model to gain insight into the mechanisms of degenerative disease, to enucleate relevant steps and to test neuroprotectants. Among them, saffron has been shown to ameliorate degenerative processes and to regulate many genes and protective pathways. Saffron has been also tested in AMD patients. We extended our analysis to a possible additional effect regulated by saffron and compared in AMD patients a pure antioxidant treatment (Lutein/zeaxanthin) with saffron treatment. **Methods:** Animal model. Sprague-Dawley (SD) adult rats, raised at 5 lux, were exposed to 1000 lux for 24 h and then either immediately sacrificed or placed back at 5 lux for 7 days recovery period. A group of animals was treated with saffron. We performed in the animal model: (1) SDS-PAGE analysis; (2) Western Blotting (3) Enzyme activity assay (4) Immunolabelling; in AMD patients: a longitudinal open-label study 29 (±5) months in two groups of patients: lutein/zeaxanthin (19) and saffron (23) treated. Visual function was tested every 8 months by ERG recordings in addition to clinical examination. **Results:** Enzymatic activity of MMP-3 is reduced in LD saffron treated retinas and is comparable to control as it is MMP-3 expression. LD treated retinas do not present “rosettes” and microglia activation and migration is highly reduced. Visual function remains stable in saffron treated AMD patients while deteriorates in the lutein/zeaxanthin group. **Conclusion:** Our results provide evidence of an additional way of action of saffron treatment confirming the complex nature of neuroprotective activities of its chemical components. Accordingly, long term follow-up in AMD patients reveals an added value of saffron supplementation treatment compared to classical antioxidant protocol.

## 1. Introduction

**Figure 1:**
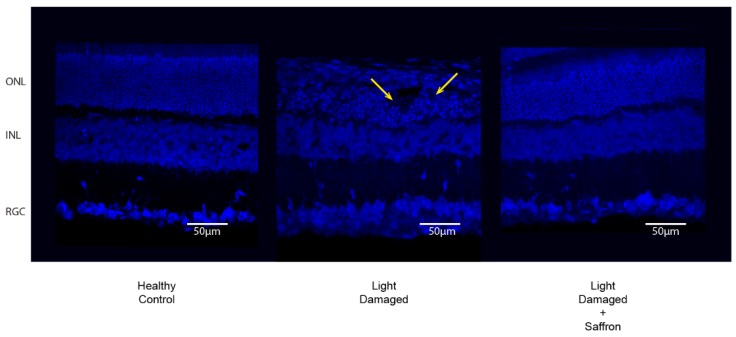
Nuclear staining (DAPI) of Control (**left**), Light damaged (**center**) and Light damaged treated with Saffron (**right**). Rosettes in LD are indicated with arrows. Images are taken in corresponding dorsal regions near the “hot spot”. ONL = outer nuclear layer; INL = inner nuclear layer; RGC = retinal ganglion cell layer.

Retinal tissue, and particularly photoreceptors, has an extremely high metabolic rate. As a consequence, the production of ROS reaches high levels in physiological conditions. A unique source of ROS generation is due to light, which is the essential stimulus to provide visual information. An efficient homeostatic system maintains the production of ROS under control [1]. With age or in pathological conditions, the equilibrium is disrupted, and a negative feedback takes place. Specifically, the reduced removal of free radicals might induce dysmetabolic processes; proteins are abnormally folded and aggregate to give rise to intra and extracellular deposits, which, in turn, produce more ROS. At this stage, an inflammatory process starts [1,2,3]. The retina presents a privileged immune-environment and a peculiar population of glial cells, specifically: (1) microglia that are resident in the inner retina and move to outer retina only in pathological conditions; (2) macroglia (astrocytes and Muller cells). Muller cells are probably modified astrocytes playing a unique role in the retina and are pivotal in reactive retinal responses to any stress. An intriguing question is when the link between oxidative stress and neuroinflammation becomes active. To understand at which level the production of ROS activates a glia response is an important issue. Even if knowledge is not complete, the sequence of events has been progressively clarified in recent papers [4,5]. The first consequence of Muller cells’ activation is the release of cytokines and trophic factors acting at different levels [6]. Microglia are activated and migrate to the outer retina in an effort to eliminate debris and support photoreceptor function. Unfortunately this event gives rise to neuroinflammatory processes that transform a supportive role of microglia into a toxic one [7,8]. Interestingly, as reported in a recent review [9], all neurodegenerative diseases (Alzheimer, Parkinson, SLA etc.) share a common feature that is the accumulation of misfolded proteins and activation of microglia. As a consequence, a vicious cycle of inflammation starts that accelerates the progression of the pathology. We used a convenient model of retinal degeneration induced by oxidative stress to understand the sequence of events and test neuro-protective agents [10,11,12]; including saffron [10,11,13]. To induce retinal neurodegeneration, albino rats were exposed to high intensity white light. This model has been validated in many experiments [10,11,12,13,14,15]. The initial event is phototoxic [16], inducing peroxidation of membrane lipids, starts in a specific dorsal area named the “hot spot” [10,14] and triggers a series of events leading to the death of photoreceptors. The degeneration progresses in time and space following the activation of a glial reaction, cytokines release, and neuroinflammation [7,8,17]. A morphological characteristic of the outer nuclear layer during the progression of the degenerative process is the presence of “rosettes” (see Figure 1). Among the genes modulated by light exposure [18], there are *matrix metalloproteinase-3* (MMP-3), *metallothionein 2A* (Mt2A), and *TIMP metalloproteinase inhibitor 1* (TIMP-1) whose activation was increased by light. Matrix metalloproteinase-3 (MMP-3) is a member of the matrix metalloproteinases, zinc-dependent endoproteases, able to degrade all the components of extracellular matrix and so could play a critical role in the dysregulation of extracellular matrix leading to the appearance of “rosettes”. Among human visual diseases, age related macular degeneration (AMD) and Stargardt, although different in their origin, share the common dependence on oxidative stress [19]. Age related macular degeneration (AMD) is a degenerative disease centered on the macula with a strong involvement of retinal pigment epithelium (RPE), Bruch’s membrane and photoreceptors. AMD is a complex disorder that affects primarily the fovea rich in cone photoreceptors. It is a late-onset disease resulting from the interplay of age and multiple genetic susceptibility genes and environmental factors, but its pathogenesis remains largely unresolved. In any case the relevance of oxidative stress and neuroinflammation in the development and progression of the disease is unquestionable. Abnormal production of free radicals initially leads to an extracellular deposit of waste molecules (drusen) in RPE that eventually induce malfunction in RPE and death of photoreceptors. Although at present the correlation between ROS production and AMD development is not completely understood, it is clear that many factors contribute to increase ROS production (see ref. [1]) and targeting free radicals for treating AMD became a must. Particularly for dry AMD, a protocol was standardized in early 2000 and gave rise to the AREDS treatment [20]. The driving idea was to use antioxidants to interfere with the progression of the disease. The original protocol was re-visited in the AREDS2 and some components of the original composition have been changed [21,22]. Nevertheless, the efficacy was verified by a long-term study showing that the supplement was able to slow down the progression of intermediate AMD. Recently natural products with antioxidant capacity have been tested. Compared to the others, saffron appeared very promising in slowing down the progression of AMD [23,24,25,26,27,28]. Interestingly, the ways of action activated by saffron are complex and not limited to a simple antioxidant activity [18,26,29,30] and it was supposed that saffron treatment might be able to activate a mechanism of tissue resilience [31], meaning that low doses and long treatment increase the probability of a positive outcome. Microarray experiments [18] have shown that saffron is able to modulate gene expression modified by retinal induced damage. Here we report data showing that saffron treatment modulates metalloproteinase expression and enzymatic activity and reduces external matrix dis-organization. These results confirm and extend our previous data [13,18,29,30] showing that the efficacy of saffron treatment is not only due to the antioxidant characteristics of its chemical components (mainly crocins) but activates complex ways of action able to modulate negative feed-back. This idea has been also tested in human subjects by a retrospective analysis performed in patients treated either with the AREDS protocol or saffron for a period of 29 months. The results confirm our hypothesis that a higher efficacy of saffron treatment is probably related to the activation of multiple pathways.

## 2. Materials and Methods

All animal experiments were authorized by the Ministry of Health (authorization number 83/96-A of 29/11/1996). In addition, all procedures were in line with the ARVO Statement for the Use of Animals in Ophthalmic and Vision Research and were approved by the local Ethical Committee of the University of L’Aquila.

**Experimental design:** A total of 50 albino rats was used for these experiments, divided in 5 experimental groups:

- Healthy control (*n* = 10) that received neither retinal damage, nor treatment;
- Light damaged no recovery group (*n* = 10) that received retinal damage but no treatment and were sacrificed immediately after light damage to perform SDS-page, western blotting, biochemical assay and MMP-3 immunostaining experiments;
- Light damaged with recovery (*n* = 10) that received retinal damage but no treatment and were sacrificed 7 days after the exposure to the light damage;
- Saffron treated + Light damaged no recovery group (*n* = 10) that received retinal damage and saffron treatment and were sacrificed immediately after light damage to perform SDS-page, western blotting, biochemical assay and MMP3 immunostaining experiments;
- Saffron treated + Light damaged with recovery (*n* = 10) that received retinal damage and saffron treatment and were sacrificed 7 days after the exposure to the light damage;

**Light damage:** Adult albino rats (2 months old) born and raised in our colony at 5 lux mean luminance were moved singularly into a cage with cold-white fluorescent lights placed at the top and at the bottom to ensure an iso-luminance environment (1000 lux) inside the cage. The litter was removed from the cage to prevent rats hiding their eyes from the light. Light exposure started at the beginning of the day in the animal house, immediately after the 12 h of darkness. Animals were consecutively exposed to 1000 lux light for 24 h and immediately placed back into normal cages and re-stabled in normal conditions for the recovery group. A group of animals was sacrificed immediately after light exposure for SDS-page, western blotting, biochemical assay and MMP-3 immunostaining.

**Saffron Treatment:** The animals belonging to the “LD + saffron” group were treated with saffron (patent: W02015/145316 which defines the best ratio among crocins) (1 mg/kg/day) soaked in tap water as diet supplementation, for 21 days before the light damage. Saffron treatment uninterruptedly continued during the whole recovery period up to the sacrifice of the animals (7 days after LD).

**Morphological Analysis:** Eyes were explanted and fixed in 4% paraformaldehyde fixative buffer at 4 °C for 24 h. After several rinses in 0.1 M phosphate-buffered saline (PBS), eyes were cryoprotected with an overnight immersion in 15% sucrose solution. After embedding with mounting medium (Tissue Tek OCT compound; Sakura Finetek, Torrance, CA, USA) and snap freezing in liquid nitrogen, cryo-sections were cut at 20 µm (CM1850 Cryostat; Leica, Wetzlar, Germany). Eye sections were collected from the center to periphery with dorsal-ventral orientation. Section were finally mounted on gelatin and poly-l-lysine coated slides.

**Immunohistochemistry:** Retinal sections were washed with 0.1 M PBS and incubated in 10% normal goat serum (Sigma, St. Louis, MO, USA) or 0.75% horse serum (Sigma, St. Louis, MO, USA) in 0.1 M PBS for 1 h at room temperature, to block non-specific binding, before incubation with the primary antibody overnight at 4 °C: Goat-MMP-3 (AbCAM, Cambridge, UK) and rabbit polyclonal IBA-1 (AbCAM, Cambridge, UK). Sections were washed in 0.1 M PBS, then incubated with the appropriate secondary antibody for 2 h at 37 °C. Immunofluorescence was viewed using a Zeiss laser scanning microscope. The data quantification represented in the figures has been collected from at least one retina section per animal for each group.

**10% SDS-PAGE**: analysis was performed on retinal protein extract according to Laemmli [32]. The protein bands were stained with Blue Coomassie R-250.

**Western Blotting:** For each sample, 30 mg protein was resolved by electrophoresis on 12% sodium dodecyl sulfate–polyacrylamide gel electrophoresis (SDS-PAGE) and was transferred onto PVDF membranes (GE Healthcare, Chicago, IL, USA). Non-specific signals were subtracted by placing blots for 1 h at room temperature incubation in a mix of 150 mM NaCl, 10 mM Tris (pH 8.0, 5% milk (Bio-rad, Hercules, CA, USA) and 0.1% Tween 20 (TBST). Membranes were incubated overnight at 4 °C with goat polyclonal secondary antibodies to MMP-3 (ab18898, AbCAM, Cambridge, UK). Membranes were washed with TBST and then incubated for 1 h at room temperature with anti–goat peroxidase-linked secondary antibodies (ab6741, AbCAM, Cambridge, UK) and with anti-rabbit peroxidase-linked secondary antibodies (sc-2030, Santa Cruz Biotechnology, Dallas, TX, USA). Protein signals were detected with a chemiluminescent reagent (ECL, West Bengal, India; Super Signal West Pico Chemiluminescent substrate; Pierce) followed by the exposure to Chemidoc (Bio-rad). Membranes were probed with an anti–α-actinin polyclonal antibody (PA5-17308, Thermo Scientific, Waltham, MA, USA) as housekeeping gene, followed by incubation with an anti–rabbit peroxidase-linked secondary antibody (sc-2030, Santa Cruz Biotechnology). Densitometric analysis was performed (Image Lab software version 4.0) on scanned files. Densitometric values obtained for MMP-3 were normalized with respect to α-actinin levels in the same blot.

**Enzyme activity:** individual rat retinas were homogenized in 200 µL of ice-buffer (Tris 20 mM, 10 µg/mL leupeptin and aprotinin, 2 mM PMSF, Triton 0.5%, pH 7.5) for 30 min; the samples were centrifuged at 15,000× *g* for 20 min and the supernatants were collected for protein concentration assay and activity measurements. MMP-3 activity was measured using a specific 5 µM MMP-3 fluorogenic substrate, MOCAc-Arg-Pro-Lys-Pro-Val-Glu-Nva-Trp-Arg-Lys(Dnp)-NH_2_ (Calbiochem) in 50 mM sodium acetate buffer, pH 5.5, for 30 min at 37 °C. The fluorescence assays were performed at 325 nm excitation and 393 nm emission using a Perkin Elmer LS50B luminescence spectrometer. The results were expressed as units of fluorescence/min·mg of total proteins.

**Statistical Analysis:** Statistical analysis was performed using the commercial package Prism 7 (Graphpad Software). In all analyses we compared Healthy, Saffron treated and Light damaged groups, applying a Two-way ANOVA test, followed by Bonferroni post-hoc tests.

**AMD patients:** Short-term clinical trial of saffron was approved by the Ethics Committee and the protocol was published in “clinicaltrials.gov” NCT00951288. Long-term clinical follow-up of AMD was approved by the Ethics Committee of the Università Cattolica (title: Clinical and genetic biomarkers of AMD) and written, informed consent was obtained from each patient after the procedures and methods of the study were fully explained. fERGs were recorded in response to a sinusoidally modulated (41 Hz) uniform field presented to the macular region (18°) at different modulations between 16.5% and 93.5% on a light-adapting background. For each fERG response, the Fourier-isolated fundamental harmonic component was measured and the noise level at this component estimated. Main outcome measures were fERG amplitude (in microV), signal-to-noise amplitude ratio (S/N in dB) and phase (in degrees).

## 3. Results

### 3.1. Light Induced Retinal Degeneration

**Figure 2:**
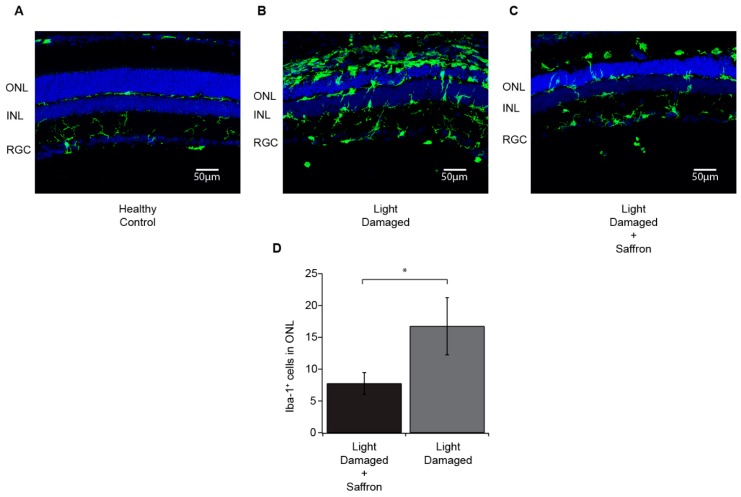
Microglia (in green) in Healthy Control (**A**), Light damaged (**B**) and Light damaged + Saffron (**C**) groups. In Healthy animals, microglia are resident in the inner retina and are absent in the outer nuclear layer (ONL) (**A**). Following light damage, resident microglia from the INL and invading microglia from the choroid migrate into the outer nuclear layer (ONL) (**B**). Saffron treatment significantly reduces the amount of microglia in the outer nuclear layer (ONL) after light damage compared to the untreated group (**C**). Number of microglia quantified, on average, in each field in the outer nuclear layer (ONL) (**D**). The mean ± SE is reported. (*p* < 0.05). Images taken in corresponding dorsal region “hot spot”. ONL = outer nuclear layer; INL = inner nuclear layer; RGC = retinal ganglion cell layer.

In agreement with previous results (see ref. [26]), light exposure induces a localized damage in the dorsal retina which is highly reduced by saffron pretreatment (Figure 1 and Figure 2). A week after the induction of damage, the progression of the neurodegenerative events is reduced as is microglia activation (Figure 2A–C). The number of Iba-positive cells in the outer nuclear layer (ONL) is significantly less in saffron treated LD retinas compared to untreated LD (Figure 2D). The width of the ONL looks preserved in agreement with a reduction in photoreceptor death as has already been shown [13] (see ref. [26]). In addition, the structure of the ONL remains highly invariant. Figure 1 shows three examples taken in corresponding retinal regions close to the “hot spot”; it is possible to note a greater number of photoreceptor layers in treated retina and the absence of “rosettes” which are clearly visible in untreated retina and are due to the dysregulation of extracellular matrix.

**Figure 3:**
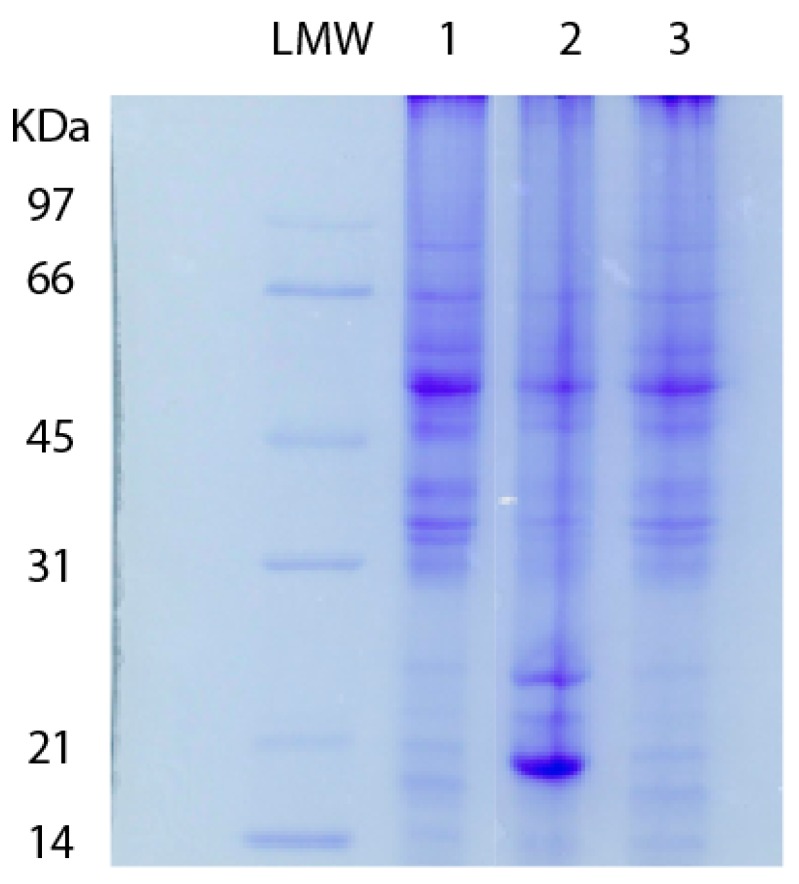
10% SDS-PAGE electrophoresis of retinal lysates: Control rats (lane 1), LD un-treated albino rats (lane 2) and saffron-treated albino rats (lane 3). Low molecular weight (LMW) standard proteins are reported on the left.

To verify the hypothesis that disorganization of extracellular matrix is strictly related to metalloprotease activity, we tested retinas explanted from LD albino rats treated and untreated with saffron according to the protocol already described [13]. We started our analysis by checking the proteolitic activity in degenerating retinas and as reported in Figure 3 we found that saffron was able to maintain protein structure in a range comparable to the healthy control. Figure 3 shows SDS-page analysis performed on retinal lysates obtained from: (1) normal control albino rat; (2) LD rat; (3) saffron treated LD rats. It can be easily noted that proteolytic activity is present in degenerating retina (line 2) after acute exposure to light. In saffron treated LD rats, the proteolitic activity is absent and a complete pattern is shown as in lane 1 (control), suggesting a maintenance in protein structure.

**Figure 4:**
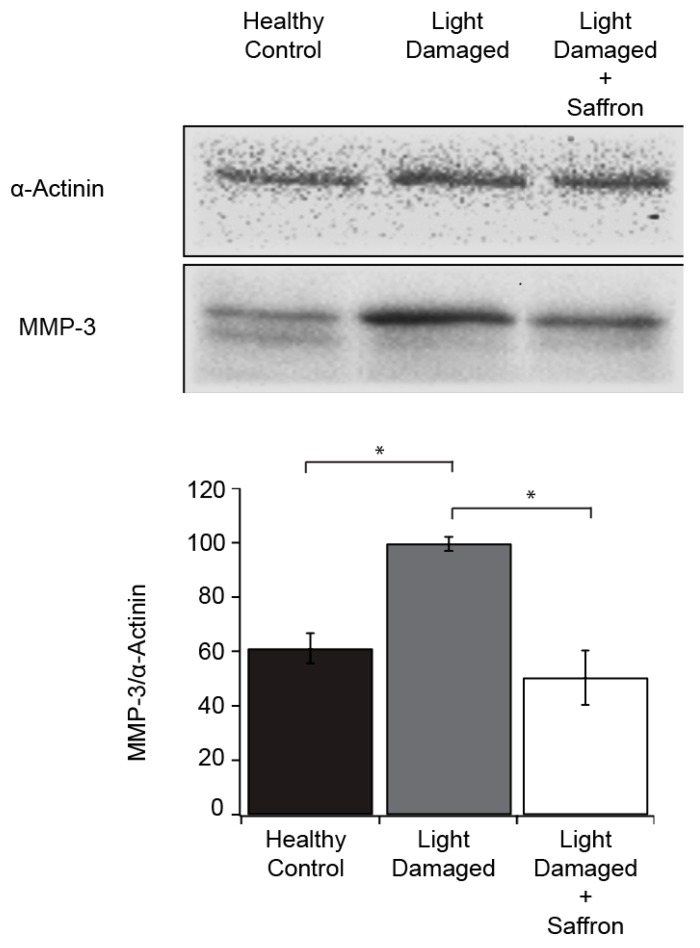
Western blot analysis of MMP-3 protein level in control, untreated and saffron treated light damaged retinas. The MMP-3 densitometric analysis normalized for α-Actinin shows that MMP-3 protein in saffron treated light damaged retinas is similar to that of control, and significantly reduced compared to the light damaged group. The mean ± SE is reported. (* = *p* < 0.05).

We performed western blot analysis as well as enzymatic activity assay and immunostaining to verify whether a relationship exists among light damage, saffron supplementation and MMP-3 regulation. Immunoblot analysis showed that MMP-3 expression was upregulated during light damage, and this increase was suppressed by treatment with saffron (Figure 4). The reduction was statistically significant.

**Figure 5:**
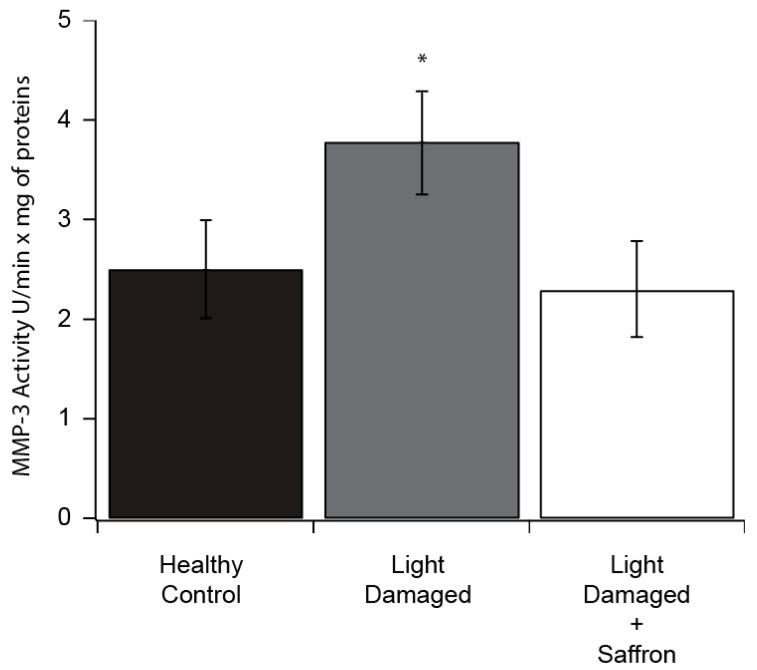
MMP-3 activity measured in retinal extracts of untreated rats (healthy control); rats exposed to light damage (Light Damaged) and rats treated with saffron and exposed to light damage (Light Damaged + Saffron). Results are expressed as the mean ± S.E of four independent experiments. * *p* < 0.02.

The enzymatic activity was tested in LD retinas of treated and untreated rats and control unstressed animals. Our data showed that in treated animals the activity of MMP-3 decreases significantly (Figure 5).

**Figure 6:**
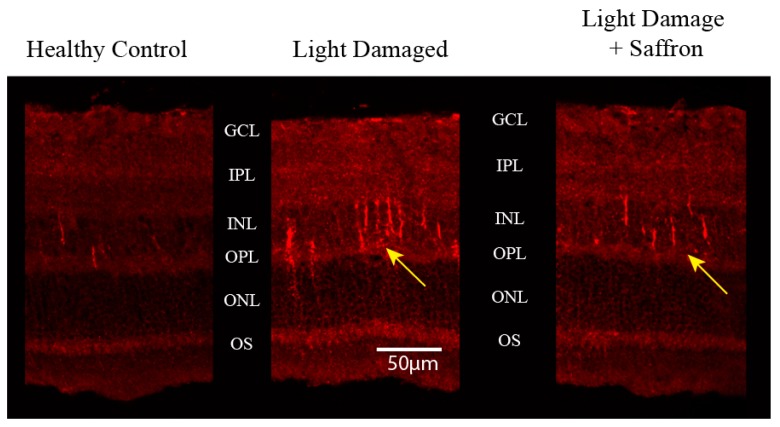
MMP-3 immunoreactivity in the different experimental models: Healthy control (**Left**), Light Damaged (**Center**) and Light Damaged with saffron supplementation (**Right**). Animals were sacrificed at the end of light exposure. OS = photoreceptor outer segments; ONL = outer nuclear layer; OPL = outer plexiform layer; INL = inner nuclear layer; IPL = inner plexiform layer; GCL = retinal ganglion cell layer.

To define the retinal localization of MMP-3, an antibody immunofluorescence analysis was performed. MMP-3 immunoreactivity was detected in thin, radial processes in the inner nuclear layer of the superior retina, likely in the Müller cells, in both groups exposed to light damage (Figure 6), but in treated animals the number of processes decreased.

### 3.2. Long-Term Treatment in AMD Patients (AREDS and Saffron)

**Figure 7:**
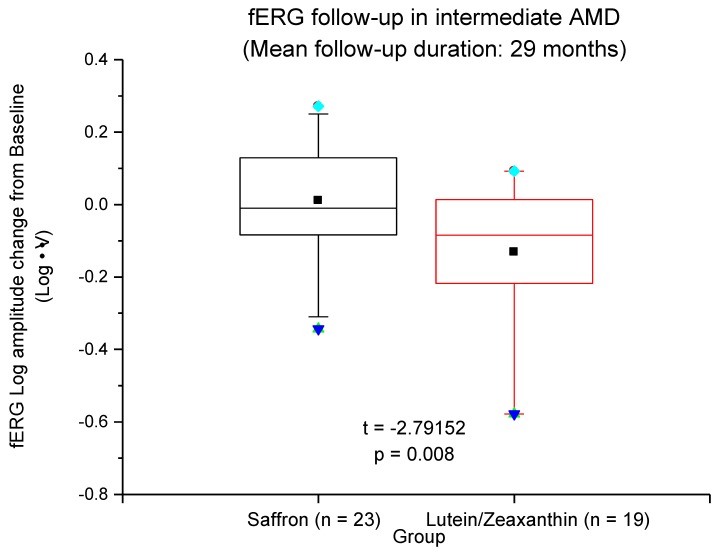
fERG Log amplitude change from baseline is reported for two groups of patients treated either with Saffron or Lutein/Zeaxanthin. Box plots showing the mean (closed squares), median (horizontal line), interquartile range (box), upper and lower 95% percentiles (error bars), upper and lower 99% percentiles (blue square and triangle, respectively) of the distribution of the fERG log amplitude changes from baseline in Saffron- and Lutein/Zeaxanthin-treated AMD patients. The difference between the two groups was highly significant (independent *t*-test).

Results obtained in two groups of patients supplemented either with Lutein/Zeaxanthin or saffron are reported in Figure 7. Visual function was measured using a standardized protocol [23]. Clinical examination and Focal (macular (18°)) electroretinogram (fERG) were recorded to estimate flicker sensitivity every 8 months over a follow-up period of 29 (±5) months. Figure 7 reports the mean change of fERG amplitude with respect to base-line. Patients treated with the AREDS protocol [20] (Lutein/Zeaxanthin) present a deterioration of retinal function whereas saffron treated patients showed quite a stable response over time; altogether these results suggest that saffron is more powerful in slowing down the progression of the disease compared to the widely employed standard AREDS supplement. Clearly, further studies on a large cohort of patients will be needed to confirm these preliminary data. Moreover, in this study, we did not include AMD patients untreated control group. We know from several previous natural history studies (i.e., [33]) that intermediate AMD may progress in a variable time span to severe visual loss. There are several risk factors that are involved, including genetics and environmental factors, and may determine the rate of progression. Therefore, a straightforward comparison between the treated patients and the historical controls or the untreated controls was not possible in the current study.

## 4. Discussion

The main results reported in this paper show that saffron treatment presents high efficiency in reducing the effects of neurodegenerative processes both in an animal model and human disease where oxidative stress and neuroinflammation are heavily involved. To our knowledge this is the first functional analysis of a comparative follow-up in two groups of AMD patients treated with two different protocols. Data obtained support the hypothesis of an added value of saffron treatment compared to the classical pure antioxidant. Results obtained in animal models and cell culture suggested this possibility. The sequence of events triggered by the production of free radical scavengers has been partially clarified and the role played by neuroinflammation has been extensively discussed [4,8,9,34] although many questions did not receive a clear answer. Accordingly, it is becoming a “must” in clinical practice to block negative feedback. Muller reactive gliosis and activation of microglia are the first signs of a retinal response to any stress and represent the first targets for neuroprotective strategies. Many therapeutic approaches have been introduced in clinical practice and saffron appeared very promising in retinal diseases associated with oxidative stress (and consequently neuroinflammation) like AMD and Stargardt. Clinical trials [23,27,28] and long term-follow up [24,25] provided evidence of the efficacy of saffron treatment in coping with AMD disease, while experiments in LD animal models and cell cultures have showed the complexity of saffron activity [10,11,13,18,26,29,30]. Data reported in this paper suggest that saffron modulates a way of action not yet explored that might cope with extracellular matrix disorganization inducing morphological features observed in both animal and human degenerating retinas. Muller cells are the first to be activated in response to any stress (oxidative in our model) and apparently, they directly control a variety of metabolic pathways including the stability of retinal tissue. They do so also by maintaining under control the expression/activation of metalloproteinases as suggested by present data. Activation of the matrix metalloproteinases is pivotal in the remodeling of the extracellular matrix and MMP-3 is usually involved in such activation mechanisms. The presence of other MMPs, namely MMP-2 and -9, in their inactive forms has been reported in the human Bruch’s membrane [35]. It can be speculated that MMP-3 released from the vesicle-like structure in the Muller glia, after light induction, could induce extra-direct proteolytic activity and/or a specific activation of other proteases such MMP-2 and -9. The analysis of the proteolyzed cleavage site fragment could give an answer to this behavior. Eventually, the stability of retinal tissue becomes compromised leading to the appearance of “rosettes”, retinal dysfunction and death. Saffron treatment looked very efficient in maintaining retinal morphology and reducing metalloproteinase activation, suggesting a new way of action in addition to the ones already clarified and the antioxidant role played directly by chemical components, mainly crocins [36]. It remains an open question whether metalloproteinase regulation and stability of retinal tissue are directly related (interdependent). Results obtained in AMD patients give support to the hypothesis of multiple mechanisms of action. Visual performance in saffron treated patients’ remains stable while deteriorates in patients treated with the AREDS protocol. The main difference between the two protocols is that AREDS is a mixture of antioxidants while saffron seems to cope also with down-stream events including neuro-inflammation, confirming the hypothesis of an added value of saffron treatment with respect to pure antioxidant treatment. Recently, Stone et al. [31] pointed out the idea of “acquired resilience” which can be generated by a tissue response to low stress levels which may induce an organized protective response. Saffron might promote resilience in response to oxidative and inflammatory stresses. A large number of clinical trials is necessary to confirm this promising result.

## 5. Conclusions

The current paper presents data supporting the hypothesis that saffron, with its complex chemical composition, may cope with neurodegenerative retinal diseases. The initial event is always multifactorial but oxidative stress plays a pivotal role at various levels including the activation of neuroinflammatory pathways. Saffron acts at different levels directly as an antioxidant but also by regulating many genes and protein synthesis. Neuronal death, neuroinflammation and morphological disorganization are reduced, and the progression of the degenerative pathology is slowed down. Data comparing two treatments in patients seems to support the hypothesis of an added value in saffron treatment.

## Abbreviations

ROS	reactive Oxygen Species
MMP-3	matrix metalloproteinase-3
Mt2A	metallothionein 2A
TIMP-1	TIMP metalloproteinase inhibitor 1
AMD	age related macular degeneration
RPE	retinal pigmented epithelium
AREDS	Age-Related Eye Disease Study
fERG	focal electroretinogram
ONL	outer nuclear layer
LD	light damaged
LMW	Low molecular weight

## References

[1] Rohowetz L., Kraus J., Koulen P. (2018). Reactive Oxygen Species-Mediated Damage of Retinal Neurons: Drug Development Targets for Therapies of Chronic Neurodegeneration of the Retina. Int. J. Mol. Sci..

[2] Moreno J.A., Gomez-Guerrero C., Mas S., Sanz A.B., Lorenzo O., Ruiz-Ortega M., Opazo L., Mezzano S., Egido J. (2018). Targeting inflammation in diabetic nephropathy: A tale of hope. Expert Opin. Investig. Drugs.

[3] Léveillard T., Philp N.J., Sennlaub F. (2019). Is Retinal Metabolic Dysfunction at the Center of the Pathogenesis of Age-related Macular Degeneration?. Int. J. Mol. Sci..

[4] Datta S., Cano M., Ebrahimi K., Wang L., Handa J.T. (2017). The impact of oxidative stress and inflammation on RPE degeneration in non-neovascular AMD. Prog. Retin. Eye Res..

[5] Rashid K., Wolf A., Langmann T. (2018). Microglia Activation and Immunomodulatory Therapies for Retinal Degenerations. Front. Cell. Neurosci..

[6] Bringmann A., Pannicke T., Grosche J., Francke M., Wiedemann P., Skatchkov S.N., Osborne N.N., Reichenbach A. (2006). Müller cells in the healthy and diseased retina. Prog. Retin. Eye Res..

[7] Vecino E., Rodriguez F.D.D., Ruzafa N., Pereiro X., Sharma S.C. (2016). Glia-neuron interactions in the mammalian retina. Prog. Retin. Eye Res..

[8] Li Q., Barres B.A. (2018). Microglia and macrophages in brain homeostasis and disease. Nat. Rev. Immunol..

[9] Guillot-Sestier M.-V., Town T. (2018). Let’s make microglia great again in neurodegenerative disorders. J. Neural Transm..

[10] Di Marco F., Di Paolo M., Romeo S., Colecchi L., Fiorani L., Spana S., Stone J., Bisti S. (2014). Combining neuroprotectants in a model of retinal degeneration: No additive benefit. PLoS ONE.

[11] Marco F.D., Romeo S., Nandasena C., Purushothuman S., Adams C., Bisti S., Stone J. (2013). The time course of action of two neuroprotectants, dietary saffron and photobiomodulation, assessed in the rat retina. Am. J. Neurodegener. Dis..

[12] Fiorani L., Passacantando M., Santucci S., Di Marco S., Bisti S., Maccarone R. (2015). Cerium Oxide Nanoparticles Reduce Microglial Activation and Neurodegenerative Events in Light Damaged Retina. PLoS ONE.

[13] Maccarone R., Di Marco S., Bisti S. (2008). Saffron supplement maintains morphology and function after exposure to damaging light in mammalian retina. Invest. Ophthalmol. Vis. Sci..

[14] Rutar M., Provis J.M., Valter K. (2010). Brief exposure to damaging light causes focal recruitment of macrophages, and long-term destabilization of photoreceptors in the albino rat retina. Curr. Eye Res..

[15] Organisciak D.T., Vaughan D.K. (2010). Retinal light damage: Mechanisms and protection. Prog. Retin. Eye Res..

[16] Demontis G.C., Longoni B., Marchiafava P.L. (2002). Molecular steps involved in light-induced oxidative damage to retinal rods. Invest. Ophthalmol. Vis. Sci..

[17] Karlstetter M., Scholz R., Rutar M., Wong W.T., Provis J.M., Langmann T. (2015). Retinal microglia: Just bystander or target for therapy?. Prog. Retin. Eye Res..

[18] Natoli R., Zhu Y., Valter K., Bisti S., Eells J., Stone J. (2010). Gene and noncoding RNA regulation underlying photoreceptor protection: Microarray study of dietary antioxidant saffron and photobiomodulation in rat retina. Mol. Vis..

[19] Maeda A., Maeda T., Golczak M., Chou S., Desai A., Hoppel C.L., Matsuyama S., Palczewski K. (2009). Involvement of all-trans-retinal in acute light-induced retinopathy of mice. J. Biol. Chem..

[20] Age-Related Eye Disease Study Research Group (1999). The Age-Related Eye Disease Study (AREDS): Design implications. AREDS report no. 1. Control. Clin. Trials.

[21] Chew E.Y., SanGiovanni J.P., Ferris F.L., Wong W.T., Agron E., Clemons T.E., Sperduto R., Danis R., Chandra S.R., Age-Related Eye Disease Study 2 (AREDS2) Research Group (2013). Lutein/zeaxanthin for the treatment of age-related cataract: AREDS2 randomized trial report no. 4. JAMA Ophthalmol..

[22] Chew E.Y., Clemons T., SanGiovanni J.P., Danis R., Domalpally A., McBee W., Sperduto R., Ferris F.L., Ferris F.L. (2012). The Age-related Eye Disease Study 2 (AREDS2). Ophthalmology.

[23] Falsini B., Piccardi M., Minnella A., Savastano C., Capoluongo E., Fadda A., Balestrazzi E., Maccarone R., Bisti S. (2010). Influence of Saffron Supplementation on Retinal Flicker Sensitivity in Early Age-Related Macular Degeneration. Invest. Ophthalmol. Vis. Sci..

[24] Piccardi M., Marangoni D., Minnella A.M., Savastano M.C., Valentini P., Ambrosio L., Capoluongo E., Maccarone R., Bisti S., Falsini B. (2012). A Longitudinal Follow-Up Study of Saffron Supplementation in Early Age-Related Macular Degeneration: Sustained Benefits to Central Retinal Function. Evidence-Based Complement. Altern. Med..

[25] Marangoni D., Falsini B., Piccardi M., Ambrosio L., Minnella A.M., Savastano M.C., Bisti S., Maccarone R., Fadda A., Mello E. (2013). Functional effect of Saffron supplementation and risk genotypes in early age-related macular degeneration: A preliminary report. J. Transl. Med..

[26] Bisti S., Maccarone R., Falsini B. (2014). Saffron and retina: Neuroprotection and pharmacokinetics. Vis. Neurosci..

[27] Riazi A., Panahi Y., Alishiri A.A., Hosseini M.A., Karimi Zarchi A.A., Sahebkar A. (2016). The impact of saffron (Crocus sativus) supplementation on visual function in patients with dry age-related macular degeneration. Ital. J. Med..

[28] Broadhead G.K., Grigg J.R., McCluskey P., Hong T., Schlub T.E., Chang A.A. (2019). Saffron therapy for the treatment of mild/moderate age-related macular degeneration: A randomised clinical trial. Graefe’s Arch. Clin. Exp. Ophthalmol..

[29] Corso L., Cavallero A., Baroni D., Garbati P., Prestipino G., Bisti S., Nobile M., Picco C. (2016). Saffron reduces ATP-induced retinal cytotoxicity by targeting P2X7 receptors. Purinergic Signal..

[30] Maccarone R., Rapino C., Zerti D., di Tommaso M., Battista N., Di Marco S., Bisti S., Maccarrone M. (2016). Modulation of Type-1 and Type-2 Cannabinoid Receptors by Saffron in a Rat Model of Retinal Neurodegeneration. PLoS ONE.

[31] Stone J., Mitrofanis J., Johnstone D.M., Falsini B., Bisti S., Adam P., Nuevo A.B., George-Weinstein M., Mason R., Eells J. (2018). Acquired Resilience: An Evolved System of Tissue Protection in Mammals. Dose-Response.

[32] Laemmli U.K. (1970). Cleavage of Structural Proteins during the Assembly of the Head of Bacteriophage T4. Nature.

[33] Chew E.Y., Clemons T.E., SanGiovanni J.P., Danis R.P., Ferris F.L., Elman M.J., Antoszyk A.N., Ruby A.J., Orth D., Bressler S.B. (2014). Secondary Analyses of the Effects of Lutein/Zeaxanthin on Age-Related Macular Degeneration Progression. JAMA Ophthalmol..

[34] Karlstetter M., Ebert S., Langmann T. (2010). Microglia in the healthy and degenerating retina: Insights from novel mouse models. Immunobiology.

[35] Liutkeviciene R., Liutkevicius V., Giedraitiene A., Kriauciuniene L., Asmoniene V., Liutkevicius V. (2017). Influence of Matrix Metalloproteinases MMP-2, -3 and on Age- Related Macular Degeneration Development. The Role of Matrix Metalloproteinase in Human Body Pathologies.

[36] Karimi E., Oskoueian E., Hendra R., Jaafar H.Z.E.E. (2010). Evaluation of Crocus sativus L. Stigma Phenolic and Flavonoid Compounds and Its Antioxidant Activity. Molecules.

